# Supplementary material for: Relationship between adolescent anemia and school attendance observed during a nationally representative survey in India
Source: Commun Med (Lond). 2024 Jun 12;4:112. doi: 10.1038/s43856-024-00533-8 (PMC11169500; doi:10.1038/s43856-024-00533-8)
Supplement: Supplementary file 2 — Supplementary Information [file 43856_2024_533_MOESM2_ESM.docx]

**Supplementary** **Information**

[Supplementary Methods 1. Description of surveys 3](#_Toc166840357)

[Supplementary Methods 2. Sensitivity analyses for the timing of exposure and outcome 6](#_Toc166840358)

[Supplementary Figure 1. Study participant flow diagram 7](#_Toc166840359)

[Supplementary Figure 2. Distribution in the reasons reported for not attending school among female out-of-school adolescents aged 15 – 18 years in India (NFHS-5, 2019-2021) 8](#_Toc166840360)

[Supplementary Table 1. Distribution in the types of relationship of the adolescent to the household head among adolescents living in households with more than one adolescent 9](#_Toc166840361)

[Supplementary Table 2. Anemia and school attendance by state and union territory (NFHS-3) 10](#_Toc166840362)

[Supplementary Table 3. Anemia and school attendance by state and union territory (NFHS-4) 11](#_Toc166840363)

[Supplementary Table 4. Anemia and school attendance by state and union territory (NFHS-5) 12](#_Toc166840364)

[Supplementary Table 5. Heterogeneity: OLS regression results for the relationship between moderate anemia and current school attendance using a separate binary predictor 13](#_Toc166840365)

[Supplementary Table 6. Heterogeneity: OLS regression results for the relationship between severe anemia and current school attendance using a separate binary predictor 14](#_Toc166840366)

[Supplementary Table 7. Heterogeneity: OLS regression results for the relationship between life-threatening anemia and current school attendance using a separate binary predictor 15](#_Toc166840367)

[Supplementary Table 8. Heterogeneity: OLS regression results for the relationship between anemia severity categories and current school attendance stratified by gender (female) 16](#_Toc166840368)

[Supplementary Table 9. Heterogeneity: OLS regression results for the relationship between anemia severity categories and current school attendance stratified by gender (male) 17](#_Toc166840369)

[Supplementary Table 10. Heterogeneity: OLS regression results for the relationship between having any anemia and current school attendance by relationship to household head 18](#_Toc166840370)

[Supplementary Table 11. Heterogeneity: OLS regression results for the relationship between having any anemia and current school attendance by household wealth quintile 19](#_Toc166840371)

[Supplementary Table 12. Heterogeneity: OLS regression results for the relationship between having any anemia and current school attendance by state-level mean school attendance 20](#_Toc166840372)

[Supplementary Table 13. Sensitivity analysis: OLS regression results for the relationship between hemoglobin concentration continuously in g/dL and current school attendance 21](#_Toc166840373)

[Supplementary Table 14. Sensitivity analyses: Household fixed-effects regression results for the relationship between having any anemia and school attendance using alternative specifications of covariates, interviewer fixed effects, sampling weights, and sample definitions 22](#_Toc166840374)

[Supplementary Table 15. Sensitivity analysis: OLS regression results for the relationship between blood transfusion as a proxy for past anemia status and school attendance 23](#_Toc166840375)

[Supplementary Table 16. Sensitivity analysis: OLS regression results for the relationship between anemia severity groups and school attendance separately by survey year (NFHS-3) 24](#_Toc166840376)

[Supplementary Table 17. Sensitivity analysis: OLS regression results for the relationship between having any anemia and school attendance separately by survey year (NFHS-4) 25](#_Toc166840377)

[Supplementary Table 18. Sensitivity analysis: OLS regression results for the relationship between having any anemia and school attendance separately by survey year (NFHS-5) 26](#_Toc166840378)

[Supplementary Table 19. Sensitivity analysis: OLS regression results for the relationship between having any anemia and selected learning outcomes available in the NFHS 2005-2019 27](#_Toc166840379)

[Supplementary References 28](#_Toc166840380)

# Supplementary Methods 1. Description of surveys

*National Family Health Survey 3*

The 2005-06 National Family Health Survey (NFHS-3) is the third in a series of household surveys which provide estimates of indicators of population, health, and nutrition by background characteristics at the national and state levels in India (IIPS 2007). The survey was conducted under the stewardship of the Ministry of Health and Family Welfare (MoHFW), Government of India, with the International Institute for Population Sciences (IIPS), Mumbai, serving as the nodal agency. ORC Macro, Calverton, Maryland, USA, provided technical assistance. NFHS-3 was funded by the United States Agency for International Development (USAID), the Department for International Development (United Kingdom), the Bill and Melinda Gates Foundation, UNICEF, the United Nations Population Fund, and the Government of India. Urban and rural samples within each state were drawn separately and, to the extent possible, the sample within each state was allocated proportionally to the size of the state’s urban and rural populations. A uniform sample design was adopted in all the states. In each state, the rural sample was selected in two stages. The selection of Primary Sampling Units (PSUs), which are villages, with probability proportional to population size (PPS) at the first stage, followed by the random selection of households within each PSU in the second stage. In urban areas, a three-stage procedure was followed. In the first stage, wards were selected with PPS sampling. In the next stage, one census enumeration block (CEB) was randomly selected from each sample ward. In the final stage, households were randomly selected within each sample CEB (IIPS 2007).

Information was collected about households, and individual interviews were conducted with women aged 15-49 and men aged 15-54. Informed consent for the survey was obtained from respondents at the start of the individual interview. NFHS-3 also included literacy and knowledge assessments as well as blood tests to ascertain anemia status. Blood specimens for anemia testing were collected by health investigators from eligible women aged 15-49 and men aged 15-54. Consent for anemia testing was taken from eligible women and men. USAID and the DHS Program interpret age 15 as the minimum age at which informed consent can be provided. Before anemia testing was undertaken, the health investigator read a detailed informed consent statement, informing that person about anemia, describing the procedure to be followed for anemia testing, and emphasizing the voluntary nature of the test (IIPS 2007). Hemoglobin levels were measured in the field using portable HemoCue Hb 201+ instruments. The HemoCue analyzer has been used extensively globally for estimating the concentration of hemoglobin in capillary blood in field situations with accurate results. HemoCue Hb 201+ uses a single drop of blood from a finger prick, which is drawn into a cuvette and then inserted into a portable, battery-operated instrument. Hemoglobin concentration was then indicated on a digital read-out. Respondents found to have severe anemia were referred to a health facility for further evaluation and treatment. Interviews were conducted with more than 124,385 women and 74,369 men. Fieldwork for NFHS-3 was conducted from December 2005 to August 2006 (IIPS 2007).

*National Family Health Survey 4*

The 2015-16 National Family Health Survey (NFHS-4) provides estimates of indicators of population, health, and nutrition by background characteristics at the national, state and district levels in India (IIPS 2017). The NFHS-4 was conducted under the stewardship of the MoHFW, Government of India. MoHFW designated the IIPS, Mumbai, as the nodal agency for the surveys. Funding for NFHS-4 was provided by USAID, the United Kingdom Department for International Development, the Bill and Melinda Gates Foundation, UNICEF, UNFPA, the MacArthur Foundation, and the Government of India. Technical assistance for NFHS-4 was provided by ICF, Maryland, USA. The rural sample was selected through a two-stage sample design with villages as the PSUs at the first stage (selected with probability proportional to size), followed by a random selection of 22 households in each PSU at the second stage. In urban areas, there was also a two-stage sample design with CEB selected at the first stage and a random selection of 22 households in each CEB at the second stage. At the second stage in both urban and rural areas, households were selected after conducting a complete mapping and household listing operation in the selected first-stage units (IIPS 2017).

Information was collected about households, and individual interviews were conducted with women aged 15-49 and men aged 15-54. Informed consent for the survey was obtained from respondents at the start of the individual interview. NFHS-3 also included literacy and knowledge assessments as well as blood tests to ascertain anemia status. Blood specimens for anemia testing were collected by health investigators from eligible women aged 15-49 and men aged 15-54. Consent for anemia testing was taken from eligible women and men. USAID and the DHS Program interpret age 15 as the minimum age at which informed consent can be provided. Before anemia testing was undertaken, the health investigator read a detailed informed consent statement, informing that person about anemia, describing the procedure to be followed for anemia testing, and emphasizing the voluntary nature of the test (IIPS 2017). Similar to the NFHS-3, hemoglobin levels were measured in the field using portable HemoCue Hb 201+ instruments. HemoCue Hb 201+ uses a single drop of blood from a finger prick, which is drawn into a cuvette and then inserted into a portable, battery-operated instrument. Hemoglobin concentration was then indicated on a digital read-out. Respondents found to have severe anemia were referred to a health facility for further evaluation and treatment. Interviews were conducted with more than 699,686 women and 103,525 men. Fieldwork was conducted from January 2015 to December 2016 (IIPS 2017).

*National Family Health Survey 5*

The 2019-21 National Family Health Survey (NFHS-5) provides essential data on health and family welfare, as well as data on emerging issues in these areas, such as levels of fertility, maternal and child health, and other health and family welfare indicators by background characteristics at the national and state levels (IIPS 2021). Like the previous NFHS, NFHS-5 was conducted under the stewardship of the MoHFW. MoHFW designated the IIPS, Mumbai, as the nodal agency for all the rounds of NFHS. Funding for NFHS-5 was provided by the MoHFW, Government of India. ICF, USA provided technical assistance through the DHS Program, which is funded by USAID. Assistance for the Dried Blood Sample (DBS) component of the survey was provided by the Indian Council of Medical Research (ICMR) and the National AIDS Research Institute (NARI), Pune. The NFHS-5 is a stratified two-stage sample. PSUs were villages in rural areas and CEBs in urban areas. The 2011 census served as the sampling frame for the selection of PSUs. Within each rural stratum, villages were selected from the sampling frame with probability proportional to size. In urban areas, CEBs were sorted according to the percentage of the scheduled castes and scheduled tribes population in each CEB, and sample CEBs were selected with PPS systematic sampling. In every selected rural and urban PSU, a complete household mapping and listing operation was conducted prior to the main survey. In the second stage, in every selected rural and urban cluster, 22 households were randomly selected with systematic sampling (IIPS 2021).

Information was collected about households, and individual interviews were conducted with women aged 15-49 and men aged 15-54. Informed consent for the survey was obtained from respondents at the start of the individual interview. NFHS-3 also included literacy and knowledge assessments as well as blood tests to ascertain anemia status. Blood specimens for anemia testing were collected by health investigators from eligible women aged 15-49 and men aged 15-54. Consent for anemia testing was taken from eligible women and men. USAID and the DHS Program interpret age 15 as the minimum age at which informed consent can be provided. Before anemia testing was undertaken, the health investigator read a detailed informed consent statement, informing that person about anemia, describing the procedure to be followed for anemia testing, and emphasizing the voluntary nature of the test (IIPS 2021). Similar to the NFHS-3 and NFHS-4, hemoglobin levels were measured in the field using portable HemoCue Hb 201+ instruments. NFHS-5 gathered information from 636,699 households, 724,115 women, and 101,839 men. In the interviewed households, 747,176 eligible women age 15-49 were identified for individual women’s interviews. Interviews were completed with 724,115 women, for a response rate of 97 percent. In all, there were 111,179 eligible men age 15-54 in households selected for the state module. Interviews were completed with 101,839 men, for a response rate of 92 percent. Fieldwork was conducted in two phases: Phase I from 17 June 2019 to 30 January 2020 covering 17 states and 5 union territories and Phase II from 2 January 2020 to 30 April 2021 covering 11 states and 3 union territories by 17 field agencies (IIPS 2021).

# Supplementary Methods 2. Sensitivity analyses for the timing of exposure and outcome

Potential misalignment between the timing of exposure and outcome may introduce bias into our results. As a first sensitivity analysis, we therefore looked at the subset of households where the time (years) between the survey and dropping out of school is short. A 16-year-old adolescent, for instance, who is not in school at the time of the survey and finished 9th grade would be more informative than the same 16-year-old who finished 5^th^ grade. To do so, we considered several specifications for time between dropping out and the survey, including the subset of households where the time between dropping out and the survey was at most one year, two years, or three years. Our main result remains consistent across all of these different specifications for the timing between school dropout and the survey (**Supplementary Table 14**).

Second, we extracted data on the reasons for not attending school among out-of-school adolescents. The basic idea for this analysis was that if anemia would precede the decision to drop out of school, adolescents may be more likely to report health-related reasons for dropping out of school as opposed to e.g., financial, or supply-side factors. Most common reported reasons for not attending school among adolescents, however, were e.g., lack of interest in studies, high costs of school, domestic activities, and marriage. Adverse health outcomes were infrequently reported in the NFHS (**Supplementary Figure 2**). Moreover, these reasons for not attending school were generally similar among out-of-school adolescents with and without any anemia.

Third, we conducted a sensitivity analysis for the timing of our exposure. To do so, we extracted data on having received a blood transfusion as a proxy for past anemia status among adolescents in the NFHS-3 (IIPS 2007), NFHS-4 (IIPS 2017), and NFHS-5 (IIPS 2021). In low- and middle-income countries, severe childhood anemia is a common indication for blood transfusions (WHO 2022). Our hypothesis was that if anemia is a true (causal) exposure of current school attendance, past anemia status as proxied by exposure to a blood transfusion would likely be associated with current school attendance. While exposure to a transfusion was related to current anemia, as expected, we found little evidence that past anemia (as proxied by exposure to a blood transfusion) was related to school attendance (**Supplementary Table 15**).

# Supplementary Figure 1. Study participant flow diagram


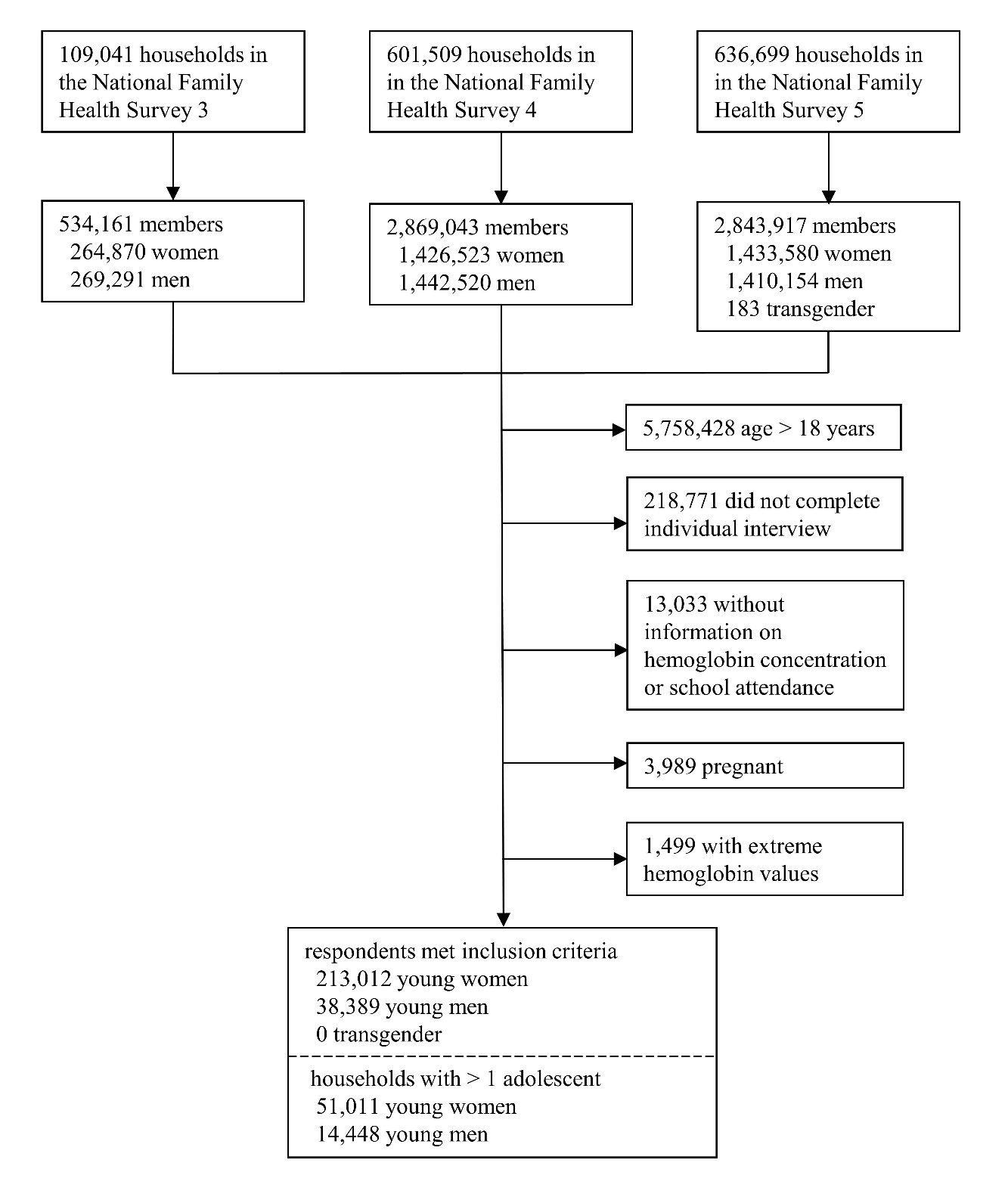


# Supplementary Figure 2. Distribution in the reasons reported for not attending school among female out-of-school adolescents aged 15 – 18 years in India (NFHS-5, 2019-2021)


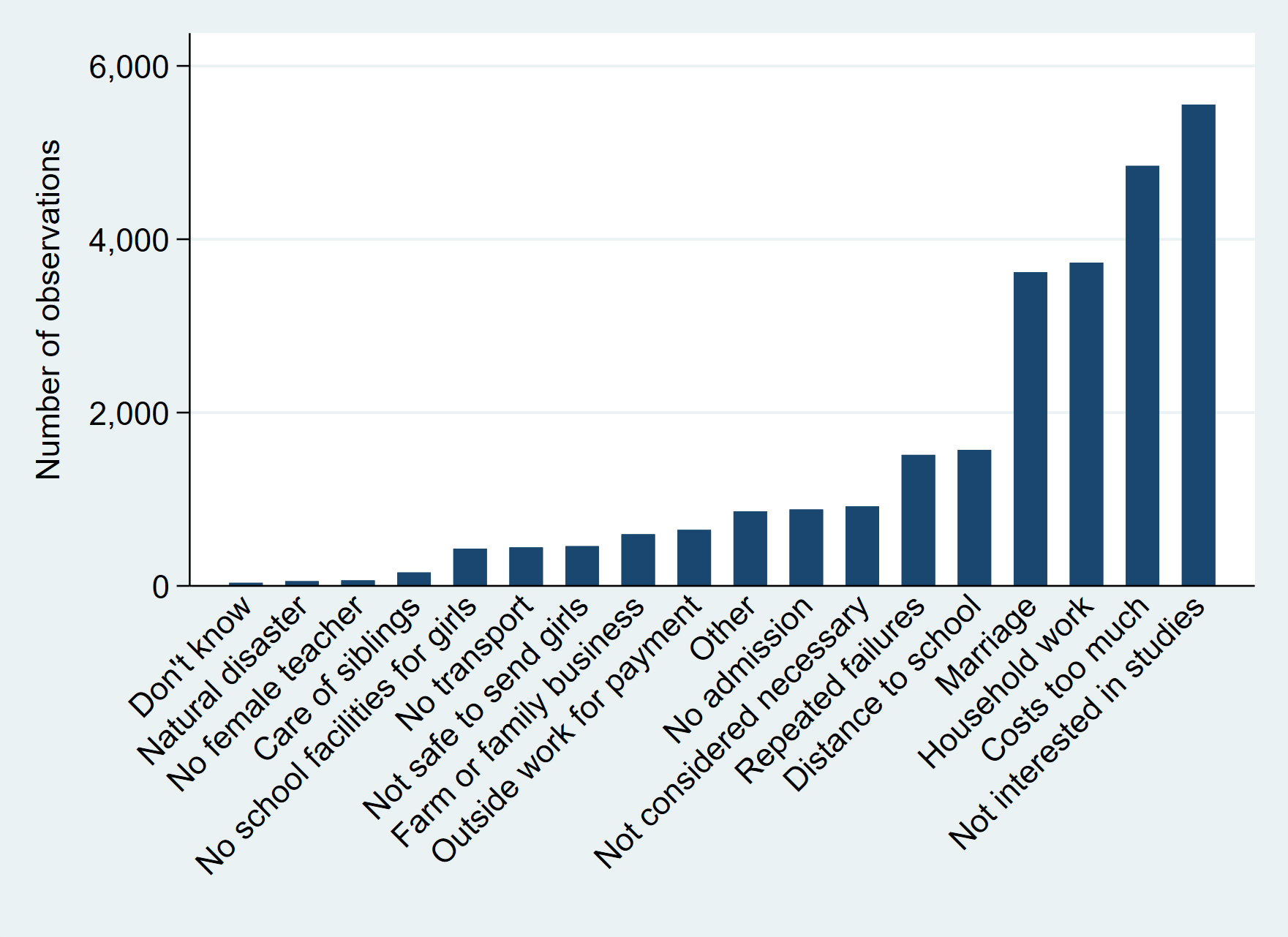


*Notes:* Figure shows the distribution in reasons for not attending school among female out-of-school adolescents in India. Sample includes all female adolescents ages 15 - 18 years old with data on measured anemia and school attendance and who were not in school at the time of the survey. Source: India NFHS-5 (*N*= 30,010). Unweighted.

# Supplementary Table 1. Distribution in the types of relationship of the adolescent to the household head among adolescents living in households with more than one adolescent

Sample includes survey respondents who were between 15 and 18 years old at the time of the survey, had a valid hemoglobin test result, and were living in a household with more than one adolescent. Source: India NFHS-3, NFHS-4, and NFHS-5.

# Supplementary Table 2. Anemia and school attendance by state and union territory (NFHS-3)

Measured anemia and school attendance among adolescents ages 15 - 18 years old.

Source: India NFHS-3 (*N*=26,839). Unweighted.

# Supplementary Table 3. Anemia and school attendance by state and union territory (NFHS-4)

Measured anemia and school attendance among adolescents ages 15 - 18 years old.

Source: India NFHS-4 (*N*=116,794). Unweighted.

# Supplementary Table 4. Anemia and school attendance by state and union territory (NFHS-5)

Measured anemia and school attendance among adolescents ages 15 - 18 years old.

Source: India NFHS-5 (*N*=107,768). Unweighted.

# Supplementary Table 5. Heterogeneity: OLS regression results for the relationship between moderate anemia and current school attendance using a separate binary predictor

*Notes:* All regressions are OLS models. The dependent variable was a binary indicator for currently attending school at the time of the survey. Standard errors in parentheses. Sample includes survey respondents who were between 15 and 18 years old at the time of the survey and had valid hemoglobin test results. No weights were used. Source: India NFHS-3, NFHS-4, and NFHS-5 (*N*=251,401).

# Supplementary Table 6. Heterogeneity: OLS regression results for the relationship between severe anemia and current school attendance using a separate binary predictor

*Notes:* All regressions are OLS models. The dependent variable was a binary indicator for currently attending school at the time of the survey. Standard errors in parentheses. Sample includes survey respondents who were between 15 and 18 years old at the time of the survey and had a valid hemoglobin test result. No weights were used. Source: India NFHS-3, NFHS-4, and NFHS-5 (*N*=251,401).

# Supplementary Table 7. Heterogeneity: OLS regression results for the relationship between life-threatening anemia and current school attendance using a separate binary predictor

*Notes:* All regressions are OLS models. The dependent variable was a binary indicator for currently attending school at the time of the survey. Standard errors in parentheses. Sample includes survey respondents who were between 15 and 18 years old at the time of the survey and had a valid hemoglobin test result. No weights were used. Source: India NFHS-3, NFHS-4, and NFHS-5 (*N*=251,401).

# Supplementary Table 8. Heterogeneity: OLS regression results for the relationship between anemia severity categories and current school attendance stratified by gender (female)

*Notes:* All regressions are ordinary least squares (OLS) models. The dependent variable was a binary indicator for currently attending school. Standard errors in parentheses. Ref: reference category. Sample includes female survey respondents who were between 15 and 18 years old at the time of the survey and had valid hemoglobin test results. No weights were used. Source: India NFHS-3, NFHS-4, and NFHS-5 (*N*=213,012).

# Supplementary Table 9. Heterogeneity: OLS regression results for the relationship between anemia severity categories and current school attendance stratified by gender (male)

*Notes:* All regressions are ordinary least squares (OLS) models. The dependent variable was a binary indicator for currently attending school. Standard errors in parentheses. Sample includes male survey respondents who were between 15 and 18 years old at the time of the survey and had valid hemoglobin test results. No weights were used. Ref: reference category. Source: India NFHS-3, NFHS-4, and NFHS-5 (*N*=251,401).

# Supplementary Table 10. Heterogeneity: OLS regression results for the relationship between having any anemia and current school attendance by relationship to household head

*Notes:* All regressions are ordinary least squares (OLS) models. The dependent variable was a binary indicator for currently attending school at the time of the survey. Standard errors in parentheses. Sample includes survey respondents who were between 15 and 18 years old at the time of the survey and had valid hemoglobin test results. No weights were used. Source: India NFHS-3, NFHS-4, and NFHS-5 (*N*=251,401).

# Supplementary Table 11. Heterogeneity: OLS regression results for the relationship between having any anemia and current school attendance by household wealth quintile

*Notes:* All regressions are ordinary least squares (OLS) models. The dependent variable was a binary indicator for currently attending school at the time of the survey. Standard errors in parentheses. Sample includes survey respondents who were between 15 and 18 years old at the time of the survey and had valid hemoglobin test results. No weights were used. Source: India NFHS-3, NFHS-4, and NFHS-5 (*N*=251,401).

# Supplementary Table 12. Heterogeneity: OLS regression results for the relationship between having any anemia and current school attendance by state-level mean school attendance

*Notes:* All regressions are ordinary least squares (OLS) models. The dependent variable was a binary indicator for currently attending school at the time of the survey. We categorized all states and union territories of India by low, medium and high level of state-level mean school attendance. Standard errors in parentheses. Sample includes survey respondents who were between 15 and 18 years old at the time of the survey and had valid hemoglobin test results. No weights were used. Source: India NFHS-3, NFHS-4, and NFHS-5 (*N*=251,401).

# Supplementary Table 13. Sensitivity analysis: OLS regression results for the relationship between hemoglobin concentration continuously in g/dL and current school attendance

*Notes:* All regressions are ordinary least squares (OLS) models. The dependent variable was a binary indicator for currently attending school at the time of the survey. Sample includes survey respondents who were between 15 and 18 years old at the time of the survey and had a valid hemoglobin test result. No weights were used. Standard errors in parentheses. Source: India NFHS-3, NFHS-4, and NFHS-5 (*N*=251,401).

# Supplementary Table 14. Sensitivity analyses: Household fixed-effects regression results for the relationship between having any anemia and school attendance using alternative specifications of covariates, interviewer fixed effects, sampling weights, and sample definitions

*Notes:* All regressions are ordinary least squares (OLS) models. The dependent variable was a binary indicator for attending school at the time of the survey. In Models 1-4, the sample includes survey respondents who were between 15 and 18 years old at the time of the survey and had a valid hemoglobin test result. In Model 5, the following out-of-school adolescents were excluded from the analysis: age 15 years old with fewer than six total years of schooling; age 16 years old with fewer than seven years of schooling; age 17 years old with fewer than eight years of schooling; and age 18 years with fewer than nine years of schooling at the time of the survey. In Model 6, the sample includes survey respondents who were between 15 and 24 years old at the time of the survey and had a valid hemoglobin test result. No weights were used except in Model 4. Standard errors in parentheses. Source: India NFHS-3, NFHS-4, and NFHS-5 (*N*=251,401).

# Supplementary Table 15. Sensitivity analysis: OLS regression results for the relationship between blood transfusion as a proxy for past anemia status and school attendance

Sample includes adolescents ages 15 – 18 years with valid hemoglobin test results and data on exposure to a blood transfusion in the past. Source: NFHS-3, NFHS-4, and NFHS-5 (*N*=247,674).

# Supplementary Table 16. Sensitivity analysis: OLS regression results for the relationship between anemia severity groups and school attendance separately by survey year (NFHS-3)

*Notes:* All regressions are ordinary least squares (OLS) models. The dependent variable was a binary indicator for currently attending school at the time of the survey. Standard errors in parentheses. Sample includes survey respondents who were between 15 and 18 years old at the time of the survey and had a valid hemoglobin test result. No weights were used. Source: India NFHS-3 (*N*= 26,839).

# Supplementary Table 17. Sensitivity analysis: OLS regression results for the relationship between having any anemia and school attendance separately by survey year (NFHS-4)

*Notes:* All regressions are ordinary least squares (OLS) models. The dependent variable was a binary indicator for currently attending school at the time of the survey. Standard errors in parentheses. Sample includes survey respondents who were between 15 and 18 years old at the time of the survey and had a valid hemoglobin test result. No weights were used. Source: India NFHS-4 (*N*=116,794).

# Supplementary Table 18. Sensitivity analysis: OLS regression results for the relationship between having any anemia and school attendance separately by survey year (NFHS-5)

*Notes:* All regressions are ordinary least squares (OLS) models. The dependent variable was a binary indicator for currently attending school at the time of the survey. Standard errors in parentheses. Sample includes survey respondents who were between 15 and 18 years old at the time of the survey and had a valid hemoglobin test result. No weights were used. Source: India NFHS-5 (*N*=107,768).

# Supplementary Table 19. Sensitivity analysis: OLS regression results for the relationship between having any anemia and selected learning outcomes available in the NFHS 2005-2019

*Notes:* All regressions are ordinary least squares (OLS) models. Panel A shows results when using more conventional covariates (no household fixed effects). Panel B includes household fixed effects in addition to pre-determined covariates. In Model 1, literacy was defined as a binary indicator with 1=having attained at least Grade 6 (NFHS-3 and NFHS-4) or Grade 9 (NFHS-5), being able to read a whole sentence or being able to read parts of a sentence, and zero otherwise (i.e., respondent cannot read at all). In Model 2, reading the news was defined as a binary indicator with 1=reading newspapers or magazines, and zero otherwise. In Model 3, comprehensive knowledge of tuberculosis was defined as 1=correctly answering five questions on the transmission and prevention of tuberculosis, and zero otherwise. In Model 4, grade progression was defined as 1=being of intended age or younger for grade completed, and zero otherwise. In Model 5, secondary school was defined as 1=having attained at least secondary school at the time of the survey, and zero otherwise. Sample includes survey respondents who were between 15 and 18 years old at the time of the survey, had valid hemoglobin test results, and were living in a household with more than one adolescent aged 15 - 18 years old. Standard errors in parentheses. Source: NFHS-3, NFHS-4, and NFHS-5 (*N*=251,401).

# Supplementary References

International Institute for Population Sciences. 2007. National Family Health Survey (NFHS-3), 2005-06: India. Mumbai, India: International Institute for Population Sciences.

International Institute for Population Sciences - IIPS/India and ICF. 2017. National Family Health Survey (NFHS-4), 2015-16: India. Mumbai: IIPS.

International Institute for Population Sciences (IIPS) and ICF. 2021. National Family Health Survey (NFHS-5), 2019-21: India. Mumbai: IIPS.

World Health Organization (2022). Blood safety and availability. [Available at: <https://www.who.int/news-room/fact-sheets/detail/blood-safety-and-availability>]
