# Supplementary material for: Relationship between adolescent anemia and school attendance observed during a nationally representative survey in India
Source: Commun Med (Lond). 2024 Jun 12;4:112. doi: 10.1038/s43856-024-00533-8 (PMC11169500; doi:10.1038/s43856-024-00533-8)
Supplement: Supplementary file 3 — Reporting Summary [file 43856_2024_533_MOESM3_ESM.pdf]

nature portfolio

Corresponding author(s): Jan-Walter De Neve

Last updated by author(s): Mar 30, 2024

Reporting Summary

Nature Portfolio wishes to improve the reproducibility of the work that we publish. This form provides structure for consistency and transparency in reporting. For further information on Nature Portfolio policies, see our Editorial Policies and the Editorial Policy Checklist.

Statistics

For all statistical analyses, confirm that the following items are present in the figure legend, table legend, main text, or Methods section.

n/a	Confirmed	
☐	☒	The exact sample size (*n*) for each experimental group/condition, given as a discrete number and unit of measurement
☐	☒	A statement on whether measurements were taken from distinct samples or whether the same sample was measured repeatedly
☒	☐	The statistical test(s) used AND whether they are one- or two-sided<br>*Only common tests should be described solely by name; describe more complex techniques in the Methods section.*
☐	☒	A description of all covariates tested
☐	☒	A description of any assumptions or corrections, such as tests of normality and adjustment for multiple comparisons
☐	☒	A full description of the statistical parameters including central tendency (e.g. means) or other basic estimates (e.g. regression coefficient) AND variation (e.g. standard deviation) or associated estimates of uncertainty (e.g. confidence intervals)
☐	☒	For null hypothesis testing, the test statistic (e.g. *F*, *t*, *r*) with confidence intervals, effect sizes, degrees of freedom and *P* value noted<br>*Give P values as exact values whenever suitable.*
☒	☐	For Bayesian analysis, information on the choice of priors and Markov chain Monte Carlo settings
☒	☐	For hierarchical and complex designs, identification of the appropriate level for tests and full reporting of outcomes
☒	☐	Estimates of effect sizes (e.g. Cohen's *d*, Pearson's *r*), indicating how they were calculated

Our web collection on statistics for biologists contains articles on many of the points above.

Software and code

Policy information about availability of computer code

Data collection	n/a
Data analysis	Stata (version 17)

For manuscripts utilizing custom algorithms or software that are central to the research but not yet described in published literature, software must be made available to editors and reviewers. We strongly encourage code deposition in a community repository (e.g. GitHub). See the Nature Portfolio guidelines for submitting code & software for further information.

Data

Policy information about availability of data

All manuscripts must include a data availability statement. This statement should provide the following information, where applicable:

- Accession codes, unique identifiers, or web links for publicly available datasets
- A description of any restrictions on data availability
- For clinical datasets or third party data, please ensure that the statement adheres to our policy

We used unrestricted data which are publicly available upon request from the Demographic and Health Surveys Program (https://dhsprogram.com/). Dataset requests must include contact information, a research project title, and a description of the proposed analysis of the data.

Human research participants

Policy information about studies involving human research participants and Sex and Gender in Research.

Reporting on sex and gender	We determined heterogeneity by gender. To do so, we interacted each anemia severity group with a male dummy in Table 3. Coefficients for the interaction between each of the anemia severity groups and our male dummy, however, were in most cases small, suggesting that the relationship between anemia and school attendance is qualitatively similar across genders. We also show results when stratifying our sample by gender (Supplementary Information).
Population characteristics	See below in "Behavioural & social sciences study design"
Recruitment	Not applicable since we used existing secondary data from the DHS.
Ethics oversight	Harvard T.H. Chan School of Public Health Institutional Review Board

Note that full information on the approval of the study protocol must also be provided in the manuscript.

Field-specific reporting

Please select the one below that is the best fit for your research. If you are not sure, read the appropriate sections before making your selection.

☐ Life sciences ☒ Behavioural & social sciences ☐ Ecological, evolutionary & environmental sciences

For a reference copy of the document with all sections, see nature.com/documents/nr-reporting-summary-flat.pdf

Behavioural & social sciences study design

All studies must disclose on these points even when the disclosure is negative.

Study description	Nationally representative household-fixed effects study using pooled cross-sectional data (quantitative).
Research sample	Data were extracted from India's 2005-06 National Family Health Survey (NFHS-3), 2015-16 National Family Health Survey (NFHS-4), and 2019-21 National Family Health Survey (NFHS-5), three cross-sectional, nationally representative household surveys with biomarker data on measured hemoglobin level and schooling outcomes. We restricted our analysis to the period of late adolescence because the NFHS measured hemoglobin for individuals aged 15 and above and secondary school attendance lasts de jury until about age 18 years, yielding a sample with an age range of 15-18 years
Sampling strategy	A total of 109,041 households were selected for the NFHS-3; a total of 601,509 households were selected for the NFHS-4; and a total of 636,699 households were selected for the NFHS-5. In the NFHS-3, all female household members aged 15–49 years and all male members aged 15–54 years were eligible to be interviewed. In the NFHS-4 and NFHS-5, all women aged 15–49 years were invited to participate while men aged 15–54 years were invited to participate in a random subsample of 15% of households. The choice to sample more women than men in the NFHS-4 and NFHS-5 was made because of the surveys' primary focus on maternal and child health. Household response rates were 98% in all three surveys (NFHS-3, NFHS-4, and NFHS-5). Individual participation rates were 95% (NFHS-3) and 97% (NFHS-4 and NFHS-5) for women, whereas individual participations rates were 87% (NFHS-3) and 92% (NFHS-4 and NFHS-5) for men. Advantages of the NFHS include high quality interviewer training, standardized data collection procedures, and consistent content over time, allowing comparability across survey years. Additional details on the NFHS surveys, including sampling and data collection, are available in Discussion 1 in the Appendix and NFHS final reports.
Data collection	Not applicable since we used existing secondary data from the DHS.
Timing	2005-2021
Data exclusions	We excluded pregnant adolescents and adolescents with extreme values of hemoglobin below 4 g/dL or above 20 g/dL under the assumption that these extreme values were likely due to measurement error. Of 269,922 adolescents aged 15-18 years who completed the surveys, 12,452 (4.6%) had a missing hemoglobin measurement, 651 (0.2%) had missing data on current school attendance, 4,164 (1.5%) reported being pregnant, and 1,519 (0.6%) had extreme hemoglobin values below 4 g/dL or above 20 g/dL, leaving a final sample for analysis of 251,401 adolescents.
Non-participation	See Figure 1 in the Appendix which shows a study participant diagram.
Randomization	Not allocated into experimental groups by researchers.

Reporting for specific materials, systems and methods

We require information from authors about some types of materials, experimental systems and methods used in many studies. Here, indicate whether each material, system or method listed is relevant to your study. If you are not sure if a list item applies to your research, read the appropriate section before selecting a response.

Materials & experimental systems

n/a	Involved in the study
☒	☐ Antibodies
☒	☐ Eukaryotic cell lines
☒	☐ Palaeontology and archaeology
☒	☐ Animals and other organisms
☒	☐ Clinical data
☒	☐ Dual use research of concern

Methods

n/a	Involved in the study
☒	☐ ChIP-seq
☒	☐ Flow cytometry
☒	☐ MRI-based neuroimaging
